# Characterization of patients receiving surgical versus non-surgical treatment for infective endocarditis in West Virginia

**DOI:** 10.1371/journal.pone.0289622

**Published:** 2023-11-14

**Authors:** Ruchi Bhandari, Noor Abdulhay, Talia Alexander, Jessica Rubenstein, Andrew Meyer, Frank H. Annie, Umar Kaleem, R. Constance Wiener, Cara Sedney, Ellen Thompson, Affan Irfan

**Affiliations:** 1 School of Public Health, West Virginia University, Morgantown, WV, United States of America; 2 National Institute for Occupational Safety and Health, Centers for Disease Control and Prevention, Atlanta, Georgia, United States of America; 3 Health Education and Research Institute, Charleston Area Medical Center, Charleston, West Virginia, United States of America; 4 Joan C. Edwards School of Medicine, Marshall University, Huntington, WV, United States of America; 5 School of Dentistry, West Virginia University, Morgantown, WV, United States of America; 6 School of Medicine, West Virginia University, Morgantown, WV, United States of America; 7 Mayo Clinic Health System, Rochester, MN, United States of America; St Paul’s Hospital Millennium Medical College, ETHIOPIA

## Abstract

**Background:**

Infective endocarditis (IE) has increased in rural states such as West Virginia (WV) with high injection drug use. IE is medically managed with antimicrobial treatment alone or combined with surgical treatment. This study aimed to characterize the predictors associated with surgical treatment and rates of inpatient mortality and readmission among IE patients in WV’s rural centers.

**Methods:**

This retrospective review of electronic health records includes all adults hospitalized for IE at major rural tertiary cardiovascular centers in WV during 2014–2018. Descriptive statistics were presented on demographics, history of injection drug use, clinical characteristics, and hospital utilization by surgery status, and multivariable logistic regression examined the association of surgery with key predictor variables, generating odds ratios (OR).

**Results:**

Of the 780 patients with IE, 38% had surgery, with a 26-fold increase in patients undergoing surgery between 2014–2018. Comparing surgery and non-surgery patients revealed significant differences. Surgery patients were significantly younger (median age 35.6 vs. 40.5 years; p<0.001); had higher rates of drug use history (80% vs. 65%; p<0.001), psychiatric disorders (57% vs. 31%; p<0.001), and readmissions (18% vs.12%; p = 0.015). Surgery patients had lower rates of discharge against medical advice (11% vs.17%; p = 0.028) and in-hospital mortality (5% vs.12%; p<0.001). In the multivariable logistic regression, surgery was associated with injection drug use (OR: 1.9; 95% CI:1.09–3. 3), indications for surgery (OR: 1.68; 95% CI:1.48–1.91), left-sided IE (OR: 2.14; 95%CI:1.43–3.19) and later years (OR:3.75; 95%CI:2.5–5.72).

**Conclusion:**

This study characterizes the predictors associated with surgical treatment and rates of inpatient mortality and readmission among IE patients across rural WV. The decision to perform cardiac surgery on IE patients is complex. Results with increased injection drug use-associated IE emphasize the importance of comprehensive care by a multidisciplinary team for optimal management of patients with IE.

## Introduction

Infective endocarditis (IE) is a severe infection of the endocardium (inner lining of the heart and/or valves) that affects 15 per 100,000 people in the United States [1], and the incidence is increasing steeply. In a recent multicenter retrospective study of electronic health records (EHR), there was an increase of 458% in patients hospitalized for IE in West Virginia (WV) during 2014–2018 [2].

IE is medically managed either with only antimicrobial treatment or a combination of antimicrobial treatment with surgical treatment [3]. The American Heart Association, the American College of Cardiology, and the European Society of Cardiology have developed evidence-based guidelines where surgical treatment is recommended in addition to antimicrobial treatment, based upon several factors, including surgical indications, heart failure and shock, microorganisms and persistent bacteremia, embolic risk reduction, right-sided IE, operative risk assessment, and risk of IE relapse [4–6].

The benefits of surgical treatment of IE under such recommendations have been demonstrated in several research studies. Surgical treatment of IE has been found to be associated with prevention of embolic sequelae and reduction of systemic embolism [6], and lower mortality in specific populations [7]. Surgery has also shown to prevent destruction of the valves and facilitate rapid recovery in patients with IE [8]. In patients who inject drugs, valve repair is preferred over replacement to preserve valve function and avoid foreign material implant [3]. However, there can be many pre- or post-operative complications in patients with injection drug use-associated IE (DU-IE), including heart failure, reinfection, neurological complications [9], stroke [10], hemodialysis, as well as prolonged respiratory failure requiring tracheostomy [8].

In the past decade, there has been a significant shift with injection drug use as the major risk factor for IE. Drug overdoses and deaths have increased sharply in the United States between 2014 and 2022, with West Virginia having the highest age-adjusted drug overdose mortality (81.4 per 100,000) [11]. Cardiac surgeries for the treatment of DU-IE have increased in tandem with the opioid epidemic in West Virginia [12]. Concomitantly, IE-related mortality has increased in new population groups, particularly in rural patients [13]. The approaches to manage and treat IE also evolve and refine with the changing risk factors [3, 14].

Given the changing epidemiology of the patient population, the purpose of this research study was to characterize predictors among patients hospitalized with IE stratified by those who received only antimicrobial treatment versus those who received antimicrobial and surgical treatment in the four major rural centers in West Virginia. We further describe the surgical characteristics and outcomes in the subpopulation of patients who received surgical treatment. This report is of particular significance because the characteristics and outcomes with respect to treatment stratification have not previously been described for patients at rural centers.

## Materials and methods

This study is a retrospective chart review of EHR of all adults between the ages of 18 to 90 years who had their first IE hospital admission between January 1, 2014, and December 31, 2018, in four university-affiliated referral hospitals in WV. The hospitals in the study are the only tertiary centers in the state with the capability to perform heart surgery, and patients are referred to these centers from across the entire state [2]. Patients were identified initially using the International Classification of Diseases, Tenth Revision, Clinical Modification (ICD-10-CM) codes for IE [2], followed by a manual chart review for all admissions to ensure accurate diagnosis confirmation. Data were captured in a secure, HIPAA-compliant, web-based system using the Research Electronic Data Capture (REDCap) [15]. During chart review, we extracted information of individual patients from history and physical examination notes, provider notes, operative notes, consultation notes, hospital narratives, laboratory tests and imaging results, and discharge summaries. The study was approved by the Institutional Review Board at West Virginia University (IRB protocol number: 1811373348). No written, signed consent was required for this retrospective study of EHR as a Health Insurance Portability and Accountability Act (HIPAA) waiver of authorization was obtained. When data were entered from EHR into REDCap, identifiable information was available. A deidentified database was created for the purpose of analyses.

The outcome variable is patients with IE undergoing surgery (along with antimicrobial treatment) vs. no surgery (antimicrobial treatment alone) at patient’s index admission for IE. Descriptive characteristics are presented on (a) demographics: sex (male/female); age (18–44, 45–64, ≥65 years); (b) substance use: smoking status (current/former/non-smoker); alcohol use (current/former/no use); injection drug use (yes/no); (c) clinical characteristics: comorbidities; number of comorbidities; psychiatric disorders; affected valve (Tricuspid/Mitral/Aortic/Pulmonic); causative organisms (methicillin-resistant *Staphylococcus aureus* [MRSA]/methicillin-susceptible *Staphylococcus aureus* [MSSA]/other); indications for surgery: valvular regurgitation (trace/mild/moderate/severe), vegetation size in each valve (diffuse thickening/small/medium/large), and embolism type; and (d) hospital utilization: consultations; length of hospital stay; length of Intensive Care Unit stay; readmission; and discharge status (alive/against medical advice (AMA)/death). The discharge categories are mutually exclusive. In addition, the following data were collected for the patients who had surgery: the valve involved (aortic, mitral, tricuspid, pulmonic), valve intervention (repair versus replacement), surgical approach (sternotomy or minimally invasive right thoracotomy), myocardial protection strategy (cardioplegia versus beating heart), and concomitant procedures.

Data were obtained for the first admission of each patient during the study period. In addition, information was also recorded on patient’s readmission during the study period. Categorical variables are presented as counts and percentages. Surgery and non-surgery groups were compared using Chi-square test or Fisher’s exact test when expected cell count was <5. Continuous variables are presented as median and interquartile range. Statistical analyses were conducted using R version 4.0.2 (R Foundation for Statistical Computing) and SPSS version 28.0. Statistical significance was accepted at *p* < 0.05. Adjustments were made using Bonferroni correction wherever multiple tests were conducted. Highlighted p-values reflect statistical significance in the tables after Bonferroni correction.

Multivariable logistic regression analysis was conducted to examine the association between the key dependent variable, surgery (yes/no), and key predictor and potentially confounding variables: age (continuous), injection drug use (dichotomous), valve (right-sided/left-sided/bilateral), number of indications for surgery (ordinal), and years (2014-2016/2017/2018). Other variables, such as sex and race, were not included in the analysis because they were not significant in the bivariate analysis.

## Results

**Fig 1:**
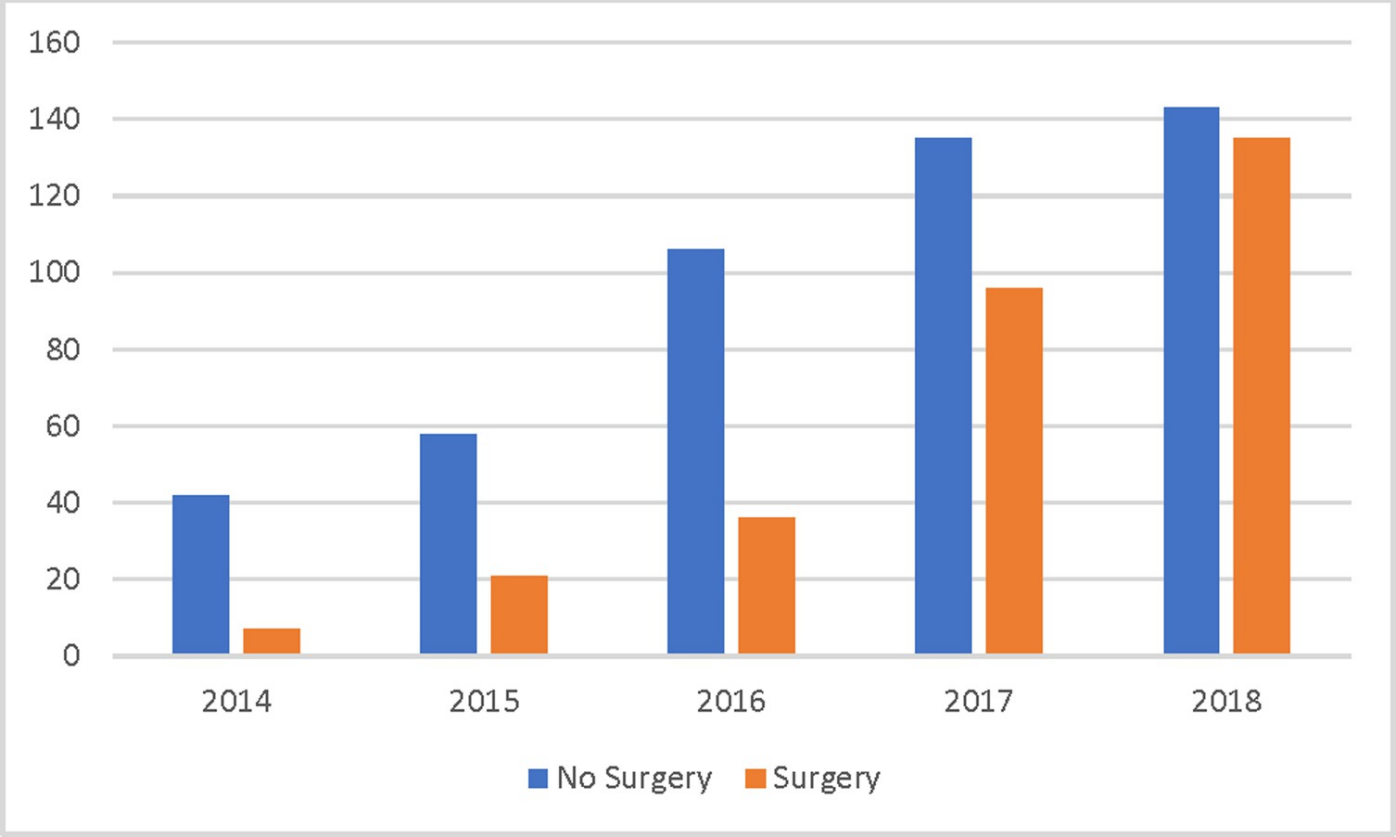
Infective endocarditis patients with and without surgery by year.

Of the 780 patients with IE who were admitted between January 1, 2014, and December 31, 2018, 37.9% had surgery. During this period, there was a 2.5-fold increase in patients who were treated non-surgically and a 26-fold increase in patients who underwent surgery as part of their management (p < 0.001) (Fig 1). The sample characteristics of the patients stratified by surgery vs. no surgery are presented in Table 1. Patients with surgery were much younger, with 71.5% in the 18–44 age group (median age of 35.6 years) compared with 58.9% of patients without surgery (median age of 40.5 years) (p < 0.001). Male and female patients hospitalized for IE during this period did not differ by surgery status. A significantly higher proportion of patients with surgery reported being current smokers (73.9% vs. 57.9%; p < 0.001), having injected drugs prior to hospital admission (80.3% vs. 65.1%; p < 0.001), and being on medications for opioid use disorder (MOUD) prior to hospital admission (33.9% vs. 16.5%; p < 0.001) compared to patients without surgery. A significantly higher proportion of patients with surgery also used opioids, amphetamines, cannabinoids, cocaine metabolites, and benzodiazepines (all p < 0.001).

**Table 1:** Baseline characteristics of patients with infective endocarditis by surgery status.

	Surgery		No Surgery		p-value
Total	N = 295	37.9%	N = 484	62.1	
	**N**	**%**	**N**	**%**	
**Years**					**< 0.001**
2014	5	1.7	41	8.5	
2015	21	7.1	58	12	
2016	36	12.2	106	21.9	
2017	96	32.5	135	27.9	
2018	135	45.8	143	29.5	
Missing	2	0.7	1	0.2	
**Sex**					0.524
Male	152	51.5	238	49.2	
Female	143	48.5	246	50.8	
**Age**					**< 0.001**
18–44	211	71.5	285	58.9	
45–64	58	19.7	114	23.6	
65+	25	8.5	85	17.6	
Missing	1	0.3	0	0	
**Age (Median and IQR)**	35.6	17.7	40.5	27.2	**< 0.001**
**Smoking status**					**< 0.001**
Current smoker	218	73.9	280	57.9	
Former smoker	47	15.9	65	13.4	
Non-smoker	22	7.5	76	15.7	
Missing	8	2.7	63	13.0	
**Alcohol use**					**0.009**
Current alcohol use	74	25.1	80	16.5	
Former alcohol use	38	12.9	30	6.2	
No alcohol use	157	53.2	251	51.9	
Missing	26	8.8	123	25.4	
**Injection drug use prior to hospital admission**					**< 0.001**
Yes	237	80.3	315	65.1	
No	57	19.3	157	32.4	
Missing	1	0.3	12	2.5	
**Type of drug**					
Opiates	203	68.8	274	56.6	**< 0.001**
Amphetamines	98	33.2	87	18.0	**< 0.001**
Cannabinoids (marijuana)	97	32.9	81	16.7	**< 0.001**
Buprenorphine	80	27.1	61	12.6	**< 0.001**
Cocaine metabolites	77	26.1	63	13.0	**< 0.001**
Benzodiazepines	53	18.0	40	8.3	**< 0.001**
Methadone	21	7.1	12	2.5	**0.002**
MOUD* prior to hospital admission					
Yes	100	33.9	80	16.5	** < 0.001**
No	139	47.1	313	64.7	
Missing	56	19.0	91	18.8	

*MOUD: Medications for opioid use disorder

Note: Highlighted p-values reflect statistical significance after Bonferroni correction.

Clinical characteristics of patients with IE stratified by surgery status are presented in Table 2. A lower proportion of patients with surgery had comorbidities, including Type 2 diabetes, coronary artery disease, hyperlipidemia, acute kidney injury, and peripheral vascular disease compared to patients without surgery. However, a higher proportion of patients with surgery were diagnosed with psychiatric disorders (57.3% vs. 31.0%) including, substance use disorder (45.1% vs. 19.0), depression (28.8% vs 15.9%), and anxiety (27.5% vs. 12.4%) (all p < 0.001), compared to patients without surgery.

**Table 2:** Clinical characteristics of patients with infective endocarditis by surgery status.

	Surgery		No Surgery		p-value
Total	N = 295	37.9%	N = 484	62.1	
	**N**	%	**N**	%	
**Comorbidities**					
Psychiatric disorders	169	57.3	150	31.0	**< 0.001**
Hypertension	82	27.8	148	30.6	0.409
Type 2 Diabetes	34	11.5	89	18.4	0.011
Coronary artery disease	22	7.5	73	15.1	**0.002**
Chronic lung disease	32	10.8	56	11.6	0.757
Hyperlipidemia	21	7.1	64	13.2	0.008
Acute kidney injury	20	6.8	57	11.8	0.023
Chronic kidney disease	22	7.5	48	9.9	0.244
Stroke	23	7.8	38	7.9	0.978
Peripheral vascular disease	13	4.4	44	9.1	0.015
Metastatic infections	11	3.7	28	5.8	0.202
Cancer	9	3.1	28	5.8	0.082
**Psychiatric disorders**					
Substance use disorder	133	45.1	92	19.0	**< 0.001**
Depression	85	28.8	77	15.9	**< 0.001**
Anxiety	81	27.5	60	12.4	**< 0.001**
Bipolar disorder	15	5.1	25	5.2	0.961
Post-traumatic stress disorder	18	6.1	17	3.5	0.091
**Type of IE**					
Tricuspid	143	48.5	209	43.2	0.150
Mitral	102	34.6	132	27.3	0.031
Aortic	91	30.8	108	22.3	**0.008**
Pulmonic	4	1.4	19	3.9	0.040
**Causative organisms**					
MRSA ‐ *Staphylococcus aureus*, methicillin resistant	106	35.9	227	46.9	**0.003**
MSSA ‐ *Staphylococcus aureus*, methicillin sensitive	88	29.8	115	23.8	0.061
*Streptococci* species	33	11.2	36	7.4	0.074
Other	20	6.8	44	9.1	0.255
Enterococcus species	27	9.2	33	6.8	0.236
Candida species	25	8.5	23	4.8	0.036
Serratia species	23	7.8	19	3.9	0.020
Culture negative	13	4.4	18	3.7	0.743
Viridians Streptococci	11	3.7	15	3.1	0.635
Klebsiella species	9	3.1	7	1.4	0.126
**Indications for surgery**					
Vegetation size	282	95.6	414	85.5	**< 0.001**
Valvular regurgitation	252	85.4	257	53.1	**< 0.001**
Embolism	163	55.3	159	32.9	**< 0.001**
Persistent sepsis/persistent positive blood cultures	108	36.6	101	20.9	**< 0.001**
Septic shock	71	24.1	75	15.5	**0.003**
Stroke	36	12.2	51	10.5	0.474
Acute congestive heart failure	27	9.2	14	2.9	**< 0.001**
Root abscess	26	8.8	6	1.2	**< 0.001**
Cardiogenic shock	13	4.4	13	2.7	0.195

Note: Highlighted p-values reflect statistical significance after Bonferroni correction.

A higher percentage of patients who underwent surgery were diagnosed with mitral valve IE (34.6% vs. 27.3%; p = 0.031) and aortic valve IE (30.8% vs. 22.3%; p = 0.008); and fewer patients with surgery had MRSA-IE (35.9% vs. 46.9%; p = 0.003), compared to patients without surgery. Additionally, more patients with surgery had the following indications for surgery: vegetation size (large ≥10mm), valvular regurgitation (mild or moderate), embolism (specifically, pulmonary embolism), persistent sepsis/persistent positive blood cultures, acute congestive heart failure, and root abscess (all p < 0.001). However, stroke and cardiogenic shock were not significantly different between the two groups.

Hospital utilization also varied between patients with and without surgical treatment, presented in Table 3. A higher proportion of patients with surgery utilized the following consultation services: cardiac surgery, general surgery, interventional radiology, dentistry, pain management, social work, physical/occupational therapy, and psychiatry. Of the patients who underwent surgery, 58.7% stayed in the hospital for 30 or more days, compared to 21% of the medically (not surgically) treated patients. Almost half (48.9%) of the patients with surgery were in the intensive care unit for six or more days compared with only 15% of the patients without surgery. Furthermore, a significantly higher proportion of patients with surgery were discharged alive (83.7% vs. 70.7%; p = 0.001); although readmissions were also higher in patients with surgery (18.3% vs. 12.0%; p = 0.015). Significantly fewer patients with surgery were discharged against medical advice (11.2 vs. 16.9%; p = 0.028), and experienced less in-hospital mortality (5.1% vs. 12.4%; p < 0.001), compared to patients without surgery.

**Table 3:** Hospital utilizations of patients with infective endocarditis by surgery status.

	Surgery		No Surgery		p-value
Total	N = 295	37.9%	N = 484	62.1	
	**N**	%	**N**	%	
**Consultations**					
Infectious Disease	291	98.6	459	94.8	**0.006**
Cardiac Surgery	289	98.0	359	74.2	**< 0.001**
Cardiology	204	69.2	344	71.1	0.569
Social work	197	66.8	190	39.3	**< 0.001**
Physical/Occupational therapy	149	50.5	171	35.3	**< 0.001**
Psychiatry	175	59.3	108	22.3	**< 0.001**
Nephrology	106	35.9	162	33.5	0.483
Dentistry	141	47.8	48	9.9	**< 0.001**
Spiritual counseling	86	29.2	90	18.6	**< 0.001**
General Surgery	74	25.1	61	12.6	**< 0.001**
Neurology	51	17.3	79	16.3	0.726
Vascular	49	16.6	65	13.4	0.223
Orthopedic Surgery	47	15.9	57	11.8	0.098
Pain management	56	19.0	19	3.9	**< 0.001**
Neurosurgery	30	10.2	45	9.3	0.689
Interventional Radiology	49	16.6	26	5.4	**< 0.001**
Ophthalmology	32	10.8	39	8.1	0.190
Individual therapy	14	4.7	4	0.8	**< 0.001**
Music therapy	5	1.7	2	0.4	0.111
**ICU length of stay**					**< 0.001**
0 hours	6	2.0	332	68.6	
>0 hours to 2 days	73	24.7	40	8.3	
3 days	30	10.2	17	3.5	
4–5 days	42	14.2	21	4.3	
6–7 days	35	11.9	15	3.1	
8–10 days	31	10.5	15	3.1	
11 or more days	77	26.1	43	8.9	
Missing	1	0.3	1	0.2	
**Discharge status**					**< 0.001**
Discharge alive	247	83.7	342	70.7	
Discharge AMA*	33	11.2	82	16.9	
Death	15	5.1	60	12.4	
**Readmission**					**0.015**
Yes	54	18.3	58	12.0	
No	241	81.7	426	88.0	
**Length of Stay**					**< 0.001 **
< = 4 days	5	1.7	70	14.5	
5–9 days	11	3.7	102	21.1	
10–19 days	11	3.7	142	29.3	
20–29 days	57	19.3	64	13.2	
30–39 days	33	11.2	29	6.0	
40–49 days	69	23.4	51	10.5	
50 or more days	71	24.1	23	4.8	
Missing	1	0.3	3	0.6	

*AMA: Against medical advice

Note: Highlighted p-values reflect statistical significance after Bonferroni correction.

The surgical characteristics of patients with IE can be observed in Table 4. Among the patients who underwent surgery, the most common EKG finding was sinus rhythm, which occurred among almost 40% of the patients. Valve replacement/repair was the predominant type of surgery, with almost half (48.6%) of the patients having a tricuspid valve replacement/repair, a third (33.9%) undergoing mitral valve replacement/repair, and about 27.8% undergoing aortic valve replacement/repair. Majority of surgeries (80%) used the median sternotomy approach with arrested heart. Almost a quarter had additional procedures such as drainage of pleural effusion and dental extractions.

**Table 4:** Surgical characteristics of patients with infective endocarditis.

	N = 295	%
**EKG Results**		
Sinus rhythm	117	39.7
Sinus tachycardia	12	4.1
Heart block	12	4.1
Permanent pacemaker required	12	4.1
**Aortic valve replacement/repair**		
Aortic valve replacement (tissue)	46	16.0
Aortic valve replacement (mechanical)	23	8.0
Aortic valve repair	11	3.8
**Mitral valve replacement/repair**		
Mitral valve repair	42	14.5
Mitral valve replacement (tissue)	42	14.5
Mitral valve replacement (mechanical)	14	4.8
**Tricuspid valve replacement/repair**		
Tricuspid valve repair	73	25.3
Tricuspid valve replacement (tissue)	61	21.2
Tricuspid valve replacement (mechanical)	6	2.1
**Surgery heart status**		
Cardioplegia	236	80.0
Beating heart (off pump)	23	7.8
Unknown	36	12.2
**Surgical approach**		
Median sternotomy	236	80.0
Right thoracotomy	21	7.1
Missing	38	12.9
**Concomitant procedures**		
Pleural effusion	74	25.1
Dental extractions (pre- or post-op)	66	22.4
Soft tissue debridement	19	6.5
Patent foramen ovale closure	16	5.4
Tracheostomy	16	5.4
Return to operating room for bleeding	15	5.1
Postop pericardial drain/window	12	4.1
Joint aspiration/debridement	14	4.7
Video-assisted thoracoscopic surgery/Thoracotomy	10	3.4

In the multivariable logistic regression results presented in Table 5, surgery was statistically significantly associated with injection drug use (OR: 1.895; 95% CI: 1.089–3.299), left-sided valve with reference to right-sided valve (2.136; 95% CI: 1.429–3.193), bilateral valve with reference to right-sided valve (2.473; 95% CI: 1.240–4.932), number of indications for surgery (OR: 1.680; 95% CI: 1.482–1.905), year 2017 with reference to 2014–2016 (OR: 3.147; 95% CI: 2.030–4.877) and year 2018 with reference to 2014–2016 (OR: 3.746; 95% CI: 2.455–5.716).

**Table 5:** Multivariable logistic regression: Surgery vs. No surgery.

Variable	Unadjusted OR	95% CI	Adjusted OR	95% CI
Age	0.981	(0.971,0.990)	0.979	(0.964, 0.995)
Injection drug use (Y/N)	2.072	(1.465, 2.931)	1.895	(1.089,3.299)
Type of IE ‐ right sided valve	Ref.	Ref.	Ref.	Ref.
Type of IE ‐ left sided valve	2.474	(1.334,4.587)	2.136	(1.429,3.193)
Type of IE ‐ bilateral valve	1.256	(0.922,1.715)	2.473	(1.240,4.932)
Indications of surgery	1.702	(1.520,1.906)	1.680	(1.482,1.905)
Year ‐ 2014–2016	Ref.	Ref.	Ref.	Ref.
Year ‐ 2017	2.351	(1.598,3.460)	3.147	(2.030,4.877)
Year ‐ 2018	3.121	(2.159,4.514)	3.746	(2.455,5.716)

## Discussion

Our results demonstrate that, compared with the number of IE patients who were only medically managed, the number of patients who were medically managed and underwent surgery increased 26-fold during the study period across four major rural centers in WV. Surgical intervention was more likely among patients who were significantly younger, injected drugs, were diagnosed with psychiatric disorders, had mitral valve IE or aortic valve IE, had more indications for surgery, and were in the intensive care unit for an extended period of time.

Findings from our study show a predominantly young population with IE, with median age of 35 years. The difference in age can be attributed to the increasing injection drug use in younger age groups in the United States. Patients who inject drugs are more likely to be younger, with fewer comorbidities or predisposing heart conditions [16]. These results are consistent with previous studies that show that the proportion of IE hospitalizations related to injection drug use has continued to increase over the years in the United States [17], and in some specific regions, such as WV [2].

Our results show a greater proportion of patients with surgery were diagnosed with psychiatric disorders, such as substance use disorder. This is not an unexpected finding, given our unique population with a high proportion of patients who had a history of injection drug use and substance use disorder [2]. Another study found significantly more DU-IE patients had valve surgery and tricuspid valve replacements compared with non-DU-IE patients [18]. Results from our study show that a higher proportion of patients who had surgery currently smoked. Smoking is thought to allow bacterial flora to enter the bloodstream through the oral tissue and can lead to the pathogenesis of infections such as IE [19]. Cigarette smoking has also been shown to redirect certain *Staphylococcus aureus* strains to virulent phenotypes associated with persistent infection [20].

Compared with patients who underwent surgery, a higher proportion of patients who were not surgically treated had comorbidities such as coronary artery disease, hyperlipidemia, acute kidney injury, and peripheral vascular disease. These results are similar to previous studies that showed higher comorbidities among patients who were not surgically treated [21, 22]. Surgery may be contraindicated in these patients due to either the severity or the number of comorbidities [22].

Our study found that compared to patients who did not have surgical treatment, significantly more patients with surgical treatment had mitral valve IE and aortic valve IE, but not tricuspid valve IE. This outcome may be due to worse hemodynamic tolerance in patients with mitral regurgitation-induced heart failure relative to tricuspid regurgitation-induced heart failure [22]. Findings from our study also show that several indications for surgery were significantly different between both groups, with more individuals who had surgery having vegetation size larger than 10 mm, pulmonary embolism, persistent sepsis/persistent positive blood cultures, or acute congestive heart failure, as is corroborated by studies [23].

Results from our study showed a significantly higher hospital utilization among surgically treated patients, both in terms of overall length of stay in the hospital and in the intensive care unit. Findings from other studies also corroborate the higher hospital utilization by patients who underwent surgical treatment [12]. However, compared to patients with surgery, more patients without surgery died during hospitalization. This finding is consistent with results from previous studies that reported higher in-hospital mortality in patients without surgical treatment [22, 24, 25]. A previous study observed the impact of early surgery in left-sided IE and found that the overall mortality was 25% at 150 weeks, and that mortality was higher in non-surgically treated patients and those that refused surgery [25]. A large national study of approximately 35,000 valve surgeries for IE found that DU-IE is associated with higher in-hospital mortality (OR 1.15, 95% CI 1.01–1.31) [26].

We found a higher rate of discharge against medical advice among patients who did not undergo surgery. Previous studies have found that patients leave the hospital against medical advice due to negative staff interactions, poor management of withdrawal or pain, boredom, or isolation [27]. However, not completing the treatment increases the risk of life-threatening infections and poorer health outcomes overall [28, 29].

In this study, we found a significantly higher proportion of patients with surgery had injected drugs prior to hospital admission, including opioids, amphetamines, cannabinoids, cocaine metabolites, and benzodiazepines. DU-IE has been associated with injection of drugs such as opioids, methamphetamines, and cocaine [14]. Patients with DU-IE frequently require longer hospital stays and have a higher chance of potentially needing a reoperation [26]. A meta-analysis demonstrated significantly worse outcomes after cardiac surgery among IE patients who inject drugs compared to patients who do not inject drugs [16]. Even the 10-year outcomes, including mortality, recurrence, and reoperation were significantly higher among DU-IE patients who underwent surgery as shown in a prospective cohort study [30].

### Public health policy

The rising DU-IE in WV has unfolded new challenges of managing, treating, and caring for the patients. Due to the complexity in diagnosis and management of IE, especially DU-IE, interprofessional collaboration is crucial in the management of patients with IE. A multidisciplinary team including specialists in infectious diseases, cardiology, cardiac surgery, microbiology, and psychiatry can help with diagnosis, management, and treatment of patients with IE [31, 32]. Previous studies have shown that a multidisciplinary team can help improve overall outcomes in patients [33, 34].

Both the treatment complexity and the numerous relevant social determinants of health that influence risk of IE render urban and rural distinctions important with respect to both outcomes and risk factors. While attention must be placed on IE in both urban and rural settings, a deeper understanding of the treatment of rural IE is key as IE is increasing at a faster rate among rural patients [35]. The high burden of DU-IE and the right-sided IE in our study are likely due to regional factors, largely driven by high prevalence of drug use. For instance, counties without syringe services programs in Kentucky saw greater increases among DU-IE than those which had syringe services programs [36]. The treatment of IE for rural patients may also differ from that for urban patients because of greater distance to care, decreased access to home health or psychiatric resources, stigma regarding drug use, or other factors [37].

### Strengths and limitations

This study has limitations. Data on demographics such as education, income, and duration of drug use were not available. While studies have shown higher mortality rates post discharge, we were only able to show mortality rates among patients with IE while they were in the hospital. A longer-term follow-up of these patients would have further strengthened the study. In addition, since we were not able to collect the timelines for the risk factors, we are restricting our interpretation to associations only. Information on drug, alcohol, and cigarette use was mostly self-reported, and likely subject to self-reporting bias. However, one noteworthy advantage of this study is that it did not rely exclusively on ICD codes to retrieve patient information, but on a complete manual chart review conducted for all patients. Another strength of this study includes its examination of individual patients rather than discharge databases.

## Conclusion

Surgery for IE has steeply increased in recent years, with the number of patients who underwent surgery increasing 26-fold between 2014 and 2018. A significantly higher number of patients with IE who had surgery were younger, currently smoked, had injected drugs and were on MOUD prior to hospital admission, were diagnosed with psychiatric disorders, and had more readmissions to hospitals as compared to those who did not have surgery. The decision to perform cardiac surgery on IE patients is complex, with outcomes varying by individual characteristics and several factors, including indications for surgery, resistance to antibiotics, prognosis of surgery, and the type of valve involved in IE. The current epidemic of injection drug use has exacerbated the need for a multidisciplinary team for comprehensive care of patients with IE.

## Supporting information

S1 Checklist STROBE statement—checklist of items that should be included in reports of observational studies. (DOCX)
