# Supplementary material for: Characterization of patients receiving surgical versus non-surgical treatment for infective endocarditis in West Virginia
Source: PLoS One. 2023 Nov 14;18(11):e0289622. doi: 10.1371/journal.pone.0289622 (PMC10645336; doi:10.1371/journal.pone.0289622)
Supplement: S1 Checklist — (DOCX) [file pone.0289622.s001.docx]

STROBE Statement—checklist of items that should be included in reports of observational studies

|  | Item No. | Recommendation | Page  No. | Relevant text from manuscript |
| --- | --- | --- | --- | --- |
| **Title and abstract** | 1 | (*a*) Indicate the study’s design with a commonly used term in the title or the abstract | 2 (abstract) | Methods: This retrospective review of electronic medical records |
|  |  | (*b*) Provide in the abstract an informative and balanced summary of what was done and what was found | 2-3 (abstract) | Methods: This retrospective review of electronic medical records includes all adults hospitalized for infective endocarditis at major rural tertiary cardiovascular centers in West Virginia from 2014 to 2018. Data were retrieved on demographics, history of drug use, clinical characteristics, and hospital utilization. Descriptive statistics were presented by surgery status, and multivariable logistic regression analysis was conducted to examine the association of surgery with key predictor variables.  Results: Of the 780 patients with infective endocarditis, 38% had surgery, with an 18-fold increase in patients undergoing surgery between 2014—2018. Compared to patients without surgery, patients who underwent surgery were significantly younger (median age 40.5 vs. 35.6 years); higher proportions had a history of drug use (65% vs. 80%), psychiatric disorders (31% vs. 57%), including substance use disorder (19% vs. 45%), and readmissions (12% vs. 18%). Significantly more patients without surgery were discharged against medical advice (17% vs. 11%) or died during hospitalization (12% vs. 5%). In the multivariable logistic regression, surgery was statistically significantly associated with the number of indications for surgery (OR: 1.74; 95% CI: 1.55—1.96), and drug use (OR: 1.73; 95% CI: 1.02—2.93). |
| Introduction | | | |  |
| Background/rationale | 2 | Explain the scientific background and rationale for the investigation being reported | 4-5 | In the past decade, there has been a significant shift with unhealthy opioid use as the major risk factor for IE. Drug overdoses and deaths have increased sharply in the United States between 2014 and 2022, with West Virginia having the highest age-adjusted drug overdose mortality (81.4 per 100,000).[11] West Virginia had a 681% increase in overall IE hospitalizations in the state between 2014 and 2018, predominantly associated with injection drug use.[2] Cardiac surgeries for the treatment of DU-IE have increased in tandem with the opioid epidemic in West Virginia.[12] Concomitantly, IE-related mortality has increased in new population groups, including rural patients in particular.[13] The approaches to manage and treat IE also evolve and refine with the changing risk factors.[14] |
| Objectives | 3 | State specific objectives, including any prespecified hypotheses | 5 | Given the changing epidemiology of the patient population, the purpose of this research study is to characterize the outcomes among patients hospitalized with IE stratified by those who received only antimicrobial treatment versus those who received antimicrobial and surgical treatment in the four major rural centers in West Virginia. We further describe the surgical characteristics and outcomes in the subpopulation of patients who received surgical treatment. This report is of particular significance because the characteristics and outcomes with respect to treatment stratification have not previously been described for patients at rural centers. |
| Methods | | | |  |
| Study design | 4 | Present key elements of study design early in the paper | 5 | This study is a retrospective chart review of electronic medical records |
| Setting | 5 | Describe the setting, locations, and relevant dates, including periods of recruitment, exposure, follow-up, and data collection | 5-6 | This study is a retrospective chart review of electronic medical records of all adults between the ages of 18 to 90 years who were hospitalized for IE at any of the four major tertiary cardiovascular centers in West Virginia between January 1, 2014, and December 31, 2018. Patients were first identified using the ICD-10 codes for IE,[2] followed by a manual chart review for all admissions. Data were captured in a secure, HIPAA-compliant, web-based system using the Research Electronic Data Capture (REDCap). Data were obtained for the first admission of each patient during the study period. |
| Participants | 6 | (*a*) *Cohort study*—Give the eligibility criteria, and the sources and methods of selection of participants. Describe methods of follow-up  *Case-control study*—Give the eligibility criteria, and the sources and methods of case ascertainment and control selection. Give the rationale for the choice of cases and controls  *Cross-sectional study*—Give the eligibility criteria, and the sources and methods of selection of participants | 5-6 | This study is a retrospective chart review of electronic medical records of all adults between the ages of 18 to 90 years who were hospitalized for IE at any of the four major tertiary cardiovascular centers in West Virginia between January 1, 2014, and December 31, 2018. Patients were first identified using the ICD-10 codes for IE,[2] followed by a manual chart review for all admissions. Data were captured in a secure, HIPAA-compliant, web-based system using the Research Electronic Data Capture (REDCap). Data were obtained for the first admission of each patient during the study period. |
|  |  | (*b*) *Cohort study*—For matched studies, give matching criteria and number of exposed and unexposed  *Case-control study*—For matched studies, give matching criteria and the number of controls per case |  |  |
| Variables | 7 | Clearly define all outcomes, exposures, predictors, potential confounders, and effect modifiers. Give diagnostic criteria, if applicable | 6-7 | Descriptive characteristics are presented on (a) demographics: sex (male/female); age; (b) substance use: smoking status (current/former/non-smoker); alcohol use (current/former/no use); drug use (yes/no); (c) clinical characteristics: comorbidities; number of comorbidities; psychiatric disorders; affected valve (Tricuspid/Mitral/Aortic/Pulmonic); causative organisms (methicillin-resistant Staphylococcus aureus [MRSA]/methicillin-susceptible Staphylococcus aureus [MSSA]/other); indications for surgery: valvular regurgitation (trace/mild/moderate/severe), vegetation size in each valve (diffuse thickening/small/medium/large), and embolism type; and (d) hospital utilization: consultations; length of hospital stay; length of Intensive Care Unit stay; readmission; and discharge status (alive/against medical advice/death). In addition, the following data were collected for the patients who had surgery: the valve involved (aortic, mitral, tricuspid, pulmonic), valve intervention (repair versus replacement), surgical approach (sternotomy or minimally invasive right thoracotomy), myocardial protection strategy (cardioplegia versus beating heart), and concomitant procedures.  Multivariable logistic regression analysis was conducted to examine the association between the key dependent variable, surgery (yes/no), and key predictor and potentially confounding variables: age (continuous), drug use (dichotomous), number of indications for surgery (ordinal), and number of comorbidities (0-2 vs. 3 or more). Other variables, such as sex and race, were not included in the analysis because they were not significant in the bivariate analysis. |
| Data sources/ measurement | 8* | For each variable of interest, give sources of data and details of methods of assessment (measurement). Describe comparability of assessment methods if there is more than one group | 6 | Patients were first identified using the ICD-10 codes for IE,[2] followed by a manual chart review for all admissions. During chart review, we extracted information of individual patients from history and physical examination notes, provider notes, operative notes, consultation notes, hospital narratives, laboratory tests and imaging results, and discharge summaries. |
| Bias | 9 | Describe any efforts to address potential sources of bias | 19 | Information on drug, alcohol, and cigarette use was mostly self-reported, and likely subject to self-reporting bias. This is a study limitation. |
| Study size | 10 | Explain how the study size was arrived at | 5 | This study is not a sample of the population – it includes all patients hospitalized for infective endocarditis during the study period. |

Continued on next page

| Quantitative variables | 11 | Explain how quantitative variables were handled in the analyses. If applicable, describe which groupings were chosen and why | 6 | Descriptive characteristics are presented on (a) demographics: sex (male/female); age (18-44, 45-64, ≥65 years; (b) substance use: smoking status (current/former/non-smoker); alcohol use (current/former/no use); drug use (yes/no); (c) clinical characteristics: comorbidities; number of comorbidities; psychiatric disorders; affected valve (Tricuspid/Mitral/Aortic/Pulmonic); causative organisms (methicillin-resistant Staphylococcus aureus [MRSA]/methicillin-susceptible Staphylococcus aureus [MSSA]/other); indications for surgery: valvular regurgitation (trace/mild/moderate/severe), vegetation size in each valve (diffuse thickening/small/medium/large), and embolism type; and (d) hospital utilization: consultations; length of hospital stay; length of Intensive Care Unit stay; readmission; and discharge status (alive/against medical advice/death). In addition, the following data were collected for the patients who had surgery: the valve involved (aortic, mitral, tricuspid, pulmonic), valve intervention (repair versus replacement), surgical approach (sternotomy or minimally invasive right thoracotomy), myocardial protection strategy (cardioplegia versus beating heart), and concomitant procedures. |
| --- | --- | --- | --- | --- |
| Statistical methods | 12 | (*a*) Describe all statistical methods, including those used to control for confounding | 6-7 | Categorical variables are presented as counts and percentages. Surgery and non-surgery groups were compared using Chi-square test or Fisher’s exact test when expected cell count was <5. Continuous variables are presented as median and interquartile range. Statistical analyses were conducted using R version 4.0.2 (R Foundation for Statistical Computing) and SPSS version 27. Statistical significance was accepted at p < 0.05. Adjustments were made using Bonferroni correction wherever multiple tests were conducted. Highlighted P-values reflect statistical significance in the tables after Bonferroni correction.  Multivariable logistic regression analysis was conducted to examine the association between the key dependent variable, surgery (yes/no), and key predictor and potentially confounding variables: age (continuous), drug use (dichotomous), number of indications for surgery (ordinal), and number of comorbidities (0-2 vs. 3 or more). Other variables, such as sex and race, were not included in the analysis because they were not significant in the bivariate analysis. |
|  |  | (*b*) Describe any methods used to examine subgroups and interactions |  |  |
|  |  | (*c*) Explain how missing data were addressed | 8-9 | Missing data are reported in Table 1 and excluded from the analyses. |
|  |  | (*d*) *Cohort study*—If applicable, explain how loss to follow-up was addressed  *Case-control study*—If applicable, explain how matching of cases and controls was addressed  *Cross-sectional study*—If applicable, describe analytical methods taking account of sampling strategy |  |  |
|  |  | (*e*) Describe any sensitivity analyses |  |  |
| Results | | | | |
| Participants | 13* | (a) Report numbers of individuals at each stage of study—eg numbers potentially eligible, examined for eligibility, confirmed eligible, included in the study, completing follow-up, and analysed | 7  8-9 | Of the 780 patients with IE who were admitted between January 1, 2014, and December 31, 2018, 37.82% had surgery.  Table 1 |
|  |  | (b) Give reasons for non-participation at each stage |  |  |
|  |  | (c) Consider use of a flow diagram |  |  |
| Descriptive data | 14* | (a) Give characteristics of study participants (eg demographic, clinical, social) and information on exposures and potential confounders | 7-8  8-9 | The sample characteristics of the patients stratified by surgery vs. no surgery are presented in Table 1. Patients with surgery were much younger, with 71.53% in the 18-44 age group (median age of 35.57 years) compared with 58.88% of patients without surgery (median age of 40.46 years) (p < 0.001). Male and female patients hospitalized for IE during this period did not differ by surgery status. Compared with patients without surgery, a significantly higher proportion of patients with surgery reported being current smokers (57.85% vs. 73.9%, p < 0.001), having used drugs prior to hospital admission (65.08% vs. 80.34%, p < 0.001), and being on medications for opioid use disorder prior to hospital admission (16.53% vs. 33.9%, p < 0.001). A significantly higher proportion of patients with surgery used opioids, amphetamines, cannabinoids, cocaine metabolites, and benzodiazepines (all p < 0.001).  Table 1 |
|  |  | (b) Indicate number of participants with missing data for each variable of interest |  |  |
|  |  | (c) *Cohort study*—Summarise follow-up time (eg, average and total amount) |  |  |
| Outcome data | 15* | *Cohort study*—Report numbers of outcome events or summary measures over time |  |  |
|  |  | *Case-control study—*Report numbers in each exposure category, or summary measures of exposure |  |  |
|  |  | *Cross-sectional study—*Report numbers of outcome events or summary measures | 10-15 | Tables 1 - 5 |
| Main results | 16 | (*a*) Give unadjusted estimates and, if applicable, confounder-adjusted estimates and their precision (eg, 95% confidence interval). Make clear which confounders were adjusted for and why they were included | 14-15 | Table 5  In the multivariable logistic regression (Table 5), surgery was statistically significantly associated with the number of indications for surgery (OR: 1.744; 95% CI: 1.550-1.962), bacteremia with MRSA/MSSA vs. other bacteria (OR: 0.689; 95% CI: 0.487-0.976), and drug use (OR: 1.732; 95% CI: 1.023-2.932). The number of comorbidities was not significantly associated with surgery in the multivariable model. |
|  |  | (*b*) Report category boundaries when continuous variables were categorized |  |  |
|  |  | (*c*) If relevant, consider translating estimates of relative risk into absolute risk for a meaningful time period |  |  |

Continued on next page

| Other analyses | 17 | Report other analyses done—eg analyses of subgroups and interactions, and sensitivity analyses |  |  |
| --- | --- | --- | --- | --- |
| Discussion | | | | |
| Key results | 18 | Summarise key results with reference to study objectives | 15 | Our results demonstrate that, compared with the number of IE patients who were only medically managed, the number of patients who were medically managed and underwent surgery increased 18-fold during the study period across four major rural centers in West Virginia. Surgical intervention was more likely among patients who were significantly younger, used drugs, were diagnosed with psychiatric disorders, had mitral valve IE or aortic valve IE, had more indications for surgery, and were in the intensive care unit for an extended period of time. |
| Limitations | 19 | Discuss limitations of the study, taking into account sources of potential bias or imprecision. Discuss both direction and magnitude of any potential bias | 19 | This study has limitations. Data on demographics such as education, income, and duration of drug use were not available. While studies have shown higher mortality rates post discharge, we were only able to show mortality rates among patients with IE while they were in the hospital. A longer-term follow-up of these patients would have further strengthened the study. Information on drug, alcohol, and cigarette use was mostly self-reported, and likely subject to self-reporting bias. |
| Interpretation | 20 | Give a cautious overall interpretation of results considering objectives, limitations, multiplicity of analyses, results from similar studies, and other relevant evidence | 19 | Surgery for IE has steeply increased in recent years, with the number of patients who underwent surgery increasing 18-fold between 2014 and 2018. A significantly higher number of patients with IE who had surgery were younger, currently smoked, had used drugs prior to hospital admissions, were diagnosed with psychiatric disorders, and had more readmissions to hospitals as compared to those who did not have surgery. The decision to perform cardiac surgery on IE patients is complex, with outcomes varying by individual characteristics and several factors, including indications for surgery, resistance to antibiotics, prognosis of surgery, type of valve involved in IE, and the current epidemic of injection drug use. A multidisciplinary team for comprehensive care of patients with IE is, therefore, critical. |
| Generalisability | 21 | Discuss the generalisability (external validity) of the study results |  |  |
| Other information | |  | | |
| Funding | 22 | Give the source of funding and the role of the funders for the present study and, if applicable, for the original study on which the present article is based | 19 | This work was supported by the National Institute of General Medical Sciences [Grant number: 2U54GM104942-07] |

*Give information separately for cases and controls in case-control studies and, if applicable, for exposed and unexposed groups in cohort and cross-sectional studies.

**Note:** An Explanation and Elaboration article discusses each checklist item and gives methodological background and published examples of transparent reporting. The STROBE checklist is best used in conjunction with this article (freely available on the Web sites of PLoS Medicine at http://www.plosmedicine.org/, Annals of Internal Medicine at http://www.annals.org/, and Epidemiology at http://www.epidem.com/). Information on the STROBE Initiative is available at www.strobe-statement.org.
