# Supplementary material for: Characterization of patients receiving surgical versus non-surgical treatment for infective endocarditis in West Virginia
Source: PLoS One. 2023 Nov 14;18(11):e0289622. doi: 10.1371/journal.pone.0289622 (PMC10645336; doi:10.1371/journal.pone.0289622)
Supplement: Attachment — Submitted filename: Response to Reviewer comments 03.27.23.docx [file pone.0289622.s002.docx]

**Response to Reviewer comments**

Thank you. I am responding to the following comments:

1. Refer to the previous submission (PONE-D-23-07172) in your cover letter
2. Upload a Response to Reviewers letter that includes a point by point response to each of the comments in your previous decision letter. This should be uploaded as a “Response to Reviewers” file type.

1. I had first submitted this manuscript on March 9 (PONE-D-23-07172) and upon the journal’s notification, I filled and submitted the STROBE checklist and the Clinical Studies Checklist. However, in the process, I missed attaching the actual IRB approval document (although the IRB protocol approval number appears in the methods section of the manuscript and I had also included it in the Clinical Studies Checklist). For this reason, the manuscript was rejected without being read by anyone. I was asked to resubmit it as a new submission, which I did on March 24.
2. In the new submission (PONE-D-23-08921), I attached the IRB approval document. I am now attaching the manuscript with track changes. I have added the following sentences in the Methods section (lines 132-136): No consent was required for this retrospective study of medical records as a Health Insurance Portability and Accountability Act (HIPAA) waiver of authorization was obtained. When data were entered from medical charts into REDCap, identifiable information was available. A deidentified database was created for the purpose of analyses.
